# KRAS G12C Mutations in NSCLC: From Target to Resistance

**DOI:** 10.3390/cancers13112541

**Published:** 2021-05-21

**Authors:** Alfredo Addeo, Giuseppe Luigi Banna, Alex Friedlaender

**Affiliations:** 1 Swiss Cancer Center Leman, Oncology Department, Switzerland University of Geneva, University Hospital Geneva, 1205 Geneva, Switzerland; afriedlaender@beaulieu.ch; 2 Oncology Department, Portsmouth Hospitals NHS Trust, Portsmouth PO2 8QD, UK; giuseppe.banna@nhs.net; 3 Oncology Service, Clinique Générale Beaulieu, 1206 Geneva, Switzerland

**Keywords:** KRAS G12C, NSCLC, sotorasib, adagrasib, resistance

## Abstract

**Simple Summary:**

A better understanding of the role of KRAS and its different mutations has led to the development of specific small-molecule inhibitors able to target KRAS G12C, an oncogenic driver mutation in a number of cancers, including non-small cell lung cancer. While these therapies hold great promise, they face the same limitation as other kinase inhibitors, the emergence of resistant mechanisms. The biology behind KRAS G12C inhibitor resistance has been investigated with genome-wide approaches, in the hopes of finding a way to improve the efficacy of these new molecules. Here, we review the biology of KRAS G12C, mechanisms of drug resistance and potential approaches to overcome the later.

**Abstract:**

Lung cancer represents the most common form of cancer, accounting for 1.8 million deaths globally in 2020. Over the last decade the treatment for advanced and metastatic non-small cell lung cancer have dramatically improved largely thanks to the emergence of two therapeutic breakthroughs: the discovery of immune checkpoint inhibitors and targeting of oncogenic driver alterations. While these therapies hold great promise, they face the same limitation as other inhibitors: the emergence of resistant mechanisms. One such alteration in non-small cell lung cancer is the Kirsten Rat Sarcoma (*KRAS*) oncogene. *KRAS* mutations are the most common oncogenic driver in NSCLC, representing roughly 20–25% of cases. The mutation is almost exclusively detected in adenocarcinoma and is found among smokers 90% of the time. Along with the development of new drugs that have been showing promising activity, resistance mechanisms have begun to be clarified. The aim of this review is to unwrap the biology of KRAS in NSCLC with a specific focus on primary and secondary resistance mechanisms and their possible clinical implications.

## 1. Introduction

Lung cancer is the most common form of cancer (in 2018, 11.6% of all new cancer cases were lung cancer cases) [1,2,3], accounting for 1.8 million deaths globally in 2020. In Europe, lung cancer was the leading cause of cancer-related deaths in 2018 representing 18.6% of these deaths. The five-year relative survival rate for lung cancer is lower than many other leading cancer types [4].

Non-small cell lung cancer (NSCLC) can be stratified into two main histotypes. The most common is lung adenocarcinoma (60%), while the second is squamous cell carcinoma (35%). These have inherent differences, in terms of causes, clinical presentation and genomic profiles [5]. Over the last decade, the treatment and prognosis of patients with advanced and metastatic NSCLC have dramatically improved largely thanks to the emergence of two therapeutic breakthroughs: the discovery of immune checkpoint inhibitors [6] and the targeting of oncogenic driver alterations. Small molecule -kinase inhibitors (KIs) targeting oncogenic mutations has led to less toxic therapeutic options, which simultaneously improved the response rates (RR) and progression-free survival (PFS), compared to platinum-based doublet chemotherapy regimens.

Kirsten Rat Sarcoma (*KRAS*) mutations are the most common oncogenic driver in NSCLC, representing roughly 20–25% of cases. Over the course of the last few years, KIs targeting a common KRAS variant, KRAS G12C, have been developed [7]. Like most oncogenic driver alterations in NSCLC, KRAS mutations are present almost exclusively in the adenocarcinoma histology [8]. However, a big difference compared to most alterations is that they are found among smokers 90% of the time [9]. Along with the development of new drugs, resistance mechanisms have begun to be elucidated.

Here, we will discuss the biology of KRAS in NSCLC, in particular KRAS G12C, with a specific focus on primary and secondary resistance mechanisms, their possible clinical implications and future research perspectives.

## 2. KRAS Biology

*RAS* proto-oncogenes are involved in coding intracellular GTPase proteins that bind to guanine nucleotides [10]. RAS proteins have two main structural components, a catalytic domain and a hypervariable region. By binding guanine nucleotides, the catalytic domain promotes activating signalling. On the other hand, the sequence of the hypervariable region influences the localization of RAS proteins on the cell membrane, impacting downstream signalling. The latter is regulated by monomeric GTPases in response to extracellular signals. The GTP-bound state leads to active signalling while the GDP-bound state leads to inactivation. The exchange between both of these states is controlled by GTPase activating proteins (GAP) their counterparts, guanine nucleotide exchange factors (GEF) [11].

GTP-bound RAS activates a number of major cellular signalling cascades. These include the RAS-RAF-MEK-ERK pathway, which regulates cell-cycles and proliferation. Another pathway involved is PI3K-AKT-mTOR, which controls cell survival. The tumour invasion and metastasis-inducing protein 1 (TIAM1-RAC1) and RAS-related protein (RAL) pathways [12] are involved in intracellular vesicle trafficking and cytoskeletal organisation and tumour growth, respectively [13].

*KRAS* mutations have been associated with tumour-promoting inflammation and play a key role in carcinogenesis by inducing an array of inflammatory cytokines, chemokines and signalling pathways that promote tumorigenesis and invasiveness [14,15].

Missense mutations leading to gains of function are implicated in oncogenesis and are clustered in a limited number of hotspots. The three most common hotspots involve codons 12, 13 and 61. Mutations in any of these codons lead to an accelerated exchange of nucleotides, and/or a decrease in the binding of GAP. Either of these increase GTP binding and KRAS activation [16].

Not all activating KRAS mutations share the same oncogenicity. Discrepancies in patient survival are associated with different Ras mutations [17,18]. In a murine model, CRISPR was used to edit genes in twelve mutations in codons 12 and 13 of KRAS. This demonstrated the heterogeneity in terms of oncogenicity of these alterations. Only five mutations ultimately led to the development of lung cancer in the mice.

Different mutations are found depending on tissue types, may confer a selective oncogenic advantage. Three factors may participate in creating the right circumstances for the development and progression of Ras-driven tumours according to each tumour type [19]. First, there is the levels of Ras proteins and the proportion of it in an activated, GTP-bound state [20]. The latter varies between 30 and 90% and is highly dependent on the KRAS mutation type [21]. Within activated forms of Ras, another factor to consider is the stability of the GTP-bound state. If there is fast-cycling, there could be less oncogenicity [16]. Furthermore, Ras levels vary between isoforms and tissue types, with up to 100 times variations. In essence, Ras signalling depends on the level of Ras proteins but also on the combination of tissue, the isoform and the mutation type. Some combinations do not lead to functional oncogenic signalling because Ras over-signalling can induce cell death or senescence without having a chance to initiate carcinogenesis [22]. The intensity of signalling and the limited functional range could be involved both in progression and resistance to targeted therapy [23].

The second factor that favours the development of Ras-driven tumours is the specificity of signalling induced by various Ras isoforms. In preclinical research, Ras isoforms stemming from a single genetic locus were found not to fully cover each other’s functions [24]. The specific signalling associated with each isoform may be influenced by distinct patterns of intracellular locations. Some may be associated with specific downstream effector pathways, influencing the impact of each isoform [25,26]. Meanwhile, in vitro analyses identified binding preferences between Ras and Raf subtypes. For instance, BRAF has a very high selectivity for KRAS, while CRAF binds and activates HRAS mediated effector MAPK pathways [27].

The third factor is the tissue and cell type. These play a role in the genetic and proteomic environments that condition Ras signalling and tumorigenesis. This environment impacts the Ras driven cell proliferation, which varies depending on the oncogene’s ability to interact with the specific drivers in the cell or tissue [28]. Each tissue type therefore has an affinity for selective Ras variants, forming the most proliferative combinations.

Through these and other minor pathways, RAS signalling regulates cellular differentiation, proliferation and apoptosis.

### RAS-Mutant Biology and Heterogeneity

The most common sites of RAS mutations are on exons 2 and 3. RAS mutations lead to GTPase function impairment and a decrease in the conversion from active, GTP-bound, to inactive, GDP-bound, RAS. As a result the downstream signalling increases. However, *KRAS* mutations are heterogeneous. Substitutions can involve codons 12, 13, or 61 [29]. The most common thereof, accounting for 40% of *KRAS* mutations in NSCLC, is the KRAS G12C. It is identified among smokers in up to 90% of the cases [30].

**Figure 1:**
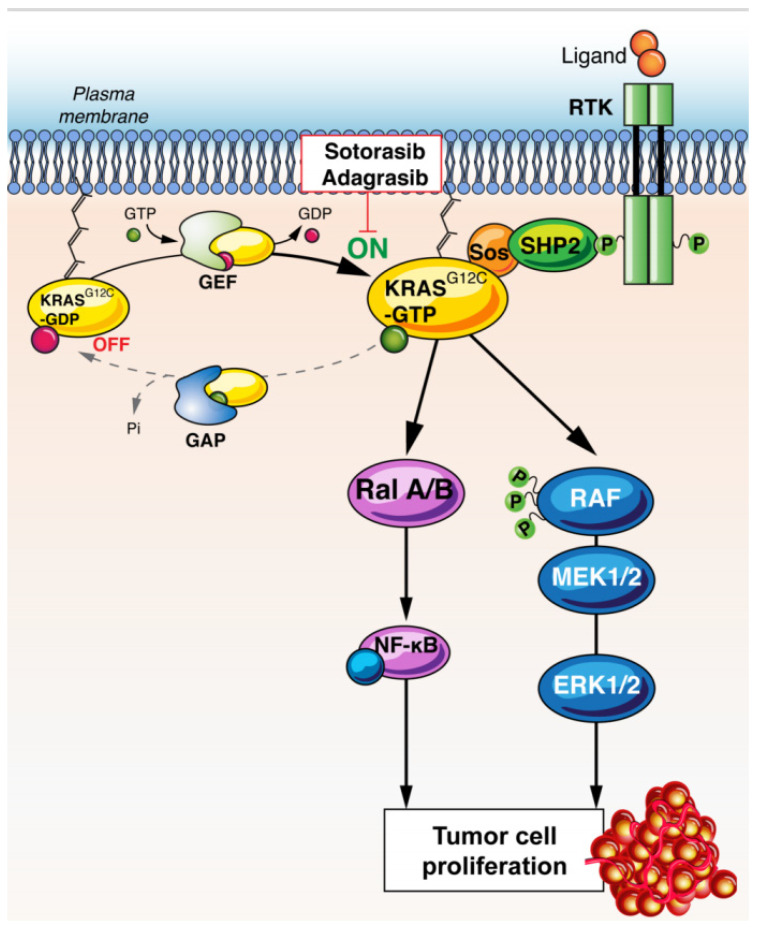
Mechanism of action of G12C inhibition. KRAS G12C signaling preferentially activates downstream Ral A/B and RAF/MEK/ERK pathways. KRAS inhibitors block these pathways, inhibiting cell proliferation. Abbreviations: RTK: receptor tyrosine-kinase, KRAS: Kirsten rat sarcoma, GAP: GTPase activating proteins, GEF: guanine nucleotide exchange factors, SOS: son of sevenless, SHP2: Src homology region 2 domain-containing phosphatase-2, Ral: Ras-like, NF-kB: nuclear factor-kB, RAF: RAF proto-oncogene serine/threonine-protein kinase, MEK: Mitogen-activated protein kinase kinase, ERK: extracellular signal-regulated kinase.

Each subtype of *KRAS* mutations has its own biology. For instance, KRAS G12, G13 and Q61 substitution mutations hinder GTP hydrolysis. On the other hand, KRAS A146T, which is the most common *KRAS* mutation in digestive cancers, has GTP-hydrolysis equivalent to that in wild-type KRAS. The KRAS A146T substitution increases nucleotide exchange, promoting the GTP-bound state. This alteration has lower oncogenic potency [7]. Each mutation activates different signalling pathways. KRAS G12C and G12V cell lines show higher RAL signalling and lower phosphorylated AKT compared to wild-type or other KRAS mutations [31] (Figure 1). Conversely, KRAS-G12D cell lines demonstrate higher levels of phosphorylated AKT, linked to preferential PI3K–AKT signalling [32,33,34,35,36].

## 3. KRAS as a Therapeutic Target

### Targeted Therapy

The first data showing clear activity with drugs targeting KRAS G12C have recently been released. Two molecules have undergone in human clinical trials and have reached phase II or III trials. These compounds rely on mutant cysteine for binding, disrupting Switch-I/II and converting KRAS preference from GTP to GDP, thus holding KRAS in the inactive GDP bound state and further inhibits RAF binding and consequent downstream signalling [37]. There are other drugs in development using a slightly different mechanism to inhibit cancer growth. Selective quinazoline-based compounds and guanosine mimetic inhibitors both suppress GTP loading of KRAS G12C, thereby hindering the signal for cell proliferation [35,38]. Allele-specific inhibition, trapping G12C in its inactive state, is another approach being developed [39,40]. Given their mechanism of action, the current direct, selective KRAS G12C KIs are not expected to result in substantial adverse events and the initial safety data are quite reassuring [41].

Sotorasib is an irreversible KRAS G12C inhibitor which locks KRAS in the GDP-bound, inactive, state. The drug has a half-life of 6 h. Preliminary results from the phase I and II sotorasib trials showed promise in terms of RR and DOR [42]. The dose escalation found 960 mg BID to be active and safe, leading to the phase II. Among NSCLC patients, the RR was 37.2%, PFS 6.3 months and DOR 10 months [43]. A phase III trial comparing docetaxel to sotorasib in patients with KRAS G12C mutation is ongoing in the second line setting. Similarly, adagrasib, another small molecule, has been tested in the phase I-II Krystal-1. Among the 51 patients in the NSCLC cohort, there was a 45% RR. Further trials are ongoing [44]. Other KRAS G12C inhibitors are under evaluation in different clinical trials (summarized in Table 1).

## 4. Mechanisms Underlying Resistance to K-Ras^G12C^ Inhibitors

**Figure 2:**
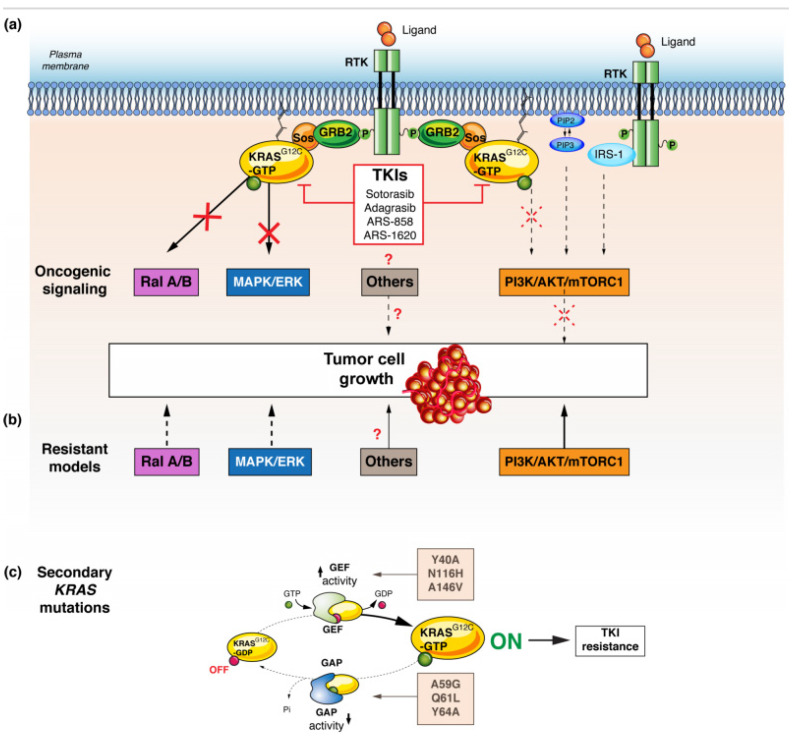
Resistance mechanisms to KRAS G12C inhibitors. (**a**) KRAS G12C inhibitors preferentially block Ral A/B and MAPK pathways. (**b**) Resistance can occur when mutant *KRAS* signaling is predominantly through the PI3K or other parallel pathways. (**c**) Secondary *KRAS* mutations confer intrinsic resistance to targeted therapy by either potentiating nucleotide exchange (secondary mutations: *Y40A*, *N116H*, or *A146V*) or impairing inherent GTPase activity (secondary mutations: *A59G*, *Q61L*, or *Y64A*). Abbreviations: RTK: receptor tyrosine-kinase, KRAS: Kirsten rat sarcoma, GAP: GTPase activating proteins, GEF: guanine nucleotide exchange factors, SOS: son of sevenless, SHP2: Src homology region 2 domain-containing phosphatase-2, Ral: Ras-like, NF-kB: nuclear factor-kB, RAF: RAF proto-oncogene serine/threonine-protein kinase, MEK: Mitogen-activated protein kinase kinase, ERK: extracellular signal-regulated kinase.

Despite the demonstrated activity of the first two KRAS G12C inhibitors, adagrasib and sotorasib, it is, unfortunately, equally clear that the vast majority of patients does not respond to them. Resistance to anticancer drugs can be either intrinsic or acquired. Given the lack of benefit in about 50–60% of patients, it is likely that certain subgroups of patients are intrinsic resistance to KRAS^G12C^ inhibitors. Causal mechanisms have not been identified in vivo and only preclinical data are available. Low dependency on KRAS signalling could confer intrinsic resistance to these inhibitors [45]. KRAS dependency varies across cell models harbouring mutant *KRAS*, meaning that some *KRAS* mutant cancers might not be driven by KRAS signaling [46]. In general, tumour cell growth is mediated by the canonical MAPK/ERK and PI3K/AKT/mTORC1 signalling pathways [47] PI3K activation is not controlled exclusively by RAS, even if the RAS protein can play an important role and interact with the PI3K p110 subunit for AKT activation [48,49]. KRAS^G12C^ inhibitors may act primarily through targeting MAPK/ERK, without affecting the phosphorylation status of AKT and mTORC1-effector pathway [39]. Therefore, parallel cell growth signalling redundancy may bypass the need for KRAS-dependent activation in cell proliferation. This could explain some inherent resistance to KRAS KIs (Figure 2a,b) [50]. Additionally, intrinsic resistance may be caused by concurrent genetic alterations that are not targeted by KRAS^G12C^ inhibitors [51]. In *KRAS^G12C^* in vitro models, secondary *KRAS* mutations confer intrinsic resistance to targeted therapy by either potentiating nucleotide exchange (secondary mutations: *Y40A*, *N116H*, or *A146V*) or impairing inherent GTPase activity (secondary mutations: *A59G*, *Q61L*, or *Y64A*) (Figure 2c) [39]. As already seen in other oncogenic mutations, the mutational status of *KRAS* gene can be heterogeneous in the same patient, leading to mixed responses to KRAS^G12C^ inhibition [52].

Every KI in the metastatic setting invariably induces resistance mechanisms. For instance, prolonged treatment of either RAF or MEK inhibitors, as used in melanoma, result in a rebound ERK activation due to the amplification of upstream drivers, such as RTKs and RAS [53]. Furthermore Xue et al. have described the mechanism of the rapid adaption of cancer cells to K-Ras^G12C^ inhibitors in cell lines and that subpopulations of *KRAS^G12C^* mutant cells respond heterogeneously to KRAS^G12C^ inhibition [54]. The adaption of cells to KRAS^G12C^ inhibition appears independent of the activity of wild-type RAS isoforms and strongly dependent on new KRAS^G12C^ production.

**Figure 3:**
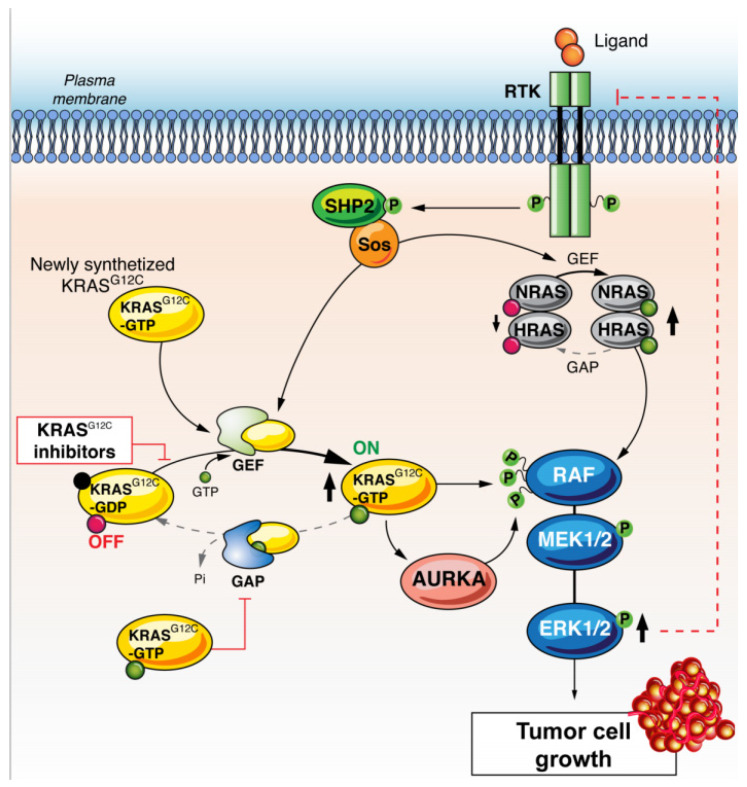
KRAS G12C inhibitor resistance through restoration of overall RAS activity due to increased RTK-SHP2 activation. Under pressure from KRAS G12C inhibitors, a feedback loop can stimulate RTKs, leading to the activation of HRAS and NRAS, and ultimately, independent KRAS G12C signalling. Abbreviations: RTK: receptor tyrosine-kinase, KRAS: Kirsten rat sarcoma, GAP: GTPase activating proteins, GEF: guanine nucleotide exchange factors, AURKA: Aurora kinase B, SOS: son of sevenless, SHP2: Src homology region 2 domain-containing phosphatase-2, Ral: Ras-like, NF-kB: nuclear factor-kB, RAF: RAF proto-oncogene serine/threonine-protein kinase, MEK: Mitogen-activated protein kinase kinase, ERK: extracellular signal-regulated kinase.

Due to this process, newly synthesized KRAS^G12C^ was maintained in its GTP-bound state to promote cancer cell proliferation. Similarly, Ryan et al. showed a similar acquired resistance pathway, with a rapid reactivation of downstream effectors after treatment with specific KRAS G12C inhibitors [55]. Increased GTP-bound wild-type RAS (N-RAS and H-RAS) proteins were responsible for restoring MAPK activation after drug treatments, even though KRAS was maintained in its inactive state. Such an intriguing difference is difficult to explain, nevertheless the 2 studies highlight that the restoration of overall RAS activity was due to increased RTK-SHP2 activation (Figure 3). To further improve KRAS G12C inhibition efficacy this acquired resistance pathway should be overcome.

Some of the intrinsic resistance to *KRAS* targeting agents identified in clinical practice as well as in preclinical models could be explained by the lack of dependency of some *KRAS* mutant tumours on *KRAS* signalling. This could stem from the differing ways in which RAS proteins activate downstream signalling. These pathways include the MAPK/ERK, as well as the PI3K/AKT/mTOR pathways. The latter’s activation does not depend solely on RAS signaling [56]. In *KRAS* mutant pancreatic ductal adenocarcinoma and lung adenocarcinoma, for example, it has been demonstrated in cell lines that the dependence on RAS signalling varies tremendously [46]. As such, even with effective complete KRAS inhibition, some *KRAS*-mutant pancreatic ductal adenocarcinoma cells survive and thrive. Upon further analysis, the majority of these cells have MAPK signalling which is PI3K-dependent, which should confer therapeutic sensitivity to inhibitors of the MAPK pathway [50]. Another mechanism allowing the bypassing of KRAS inhibition in preclinical cancer cell lines is the amplification of a transcriptional coactivator, YAP1 [57].

In addition to the diverse mechanisms of intrinsic resistance to KRAS targeting drugs, acquired resistance frequently emerges. In the era before direct KRAS inhibitors, this phenomenon was often associated with resistance to therapy targeting various steps of the downstream signalling pathway [11]. MEK is among the most common targets in the MAPK pathway, but MEK inhibitors have been largely disappointing in this context, proving to be of a very limited activity in patients with lung adenocarcinoma harbouring *KRAS* mutations [58]. Given the early promise of finally targeting KRAS indirectly through this approach, there was a randomised controlled trial, the SELECT-1 trial, which compared docetaxel alone to a combination with selumetinib, a MEK inhibitor in patients with KRAS-mutant lung adenocarcinoma. In spite of the large study population of 510 patients, there was no significant difference in either progression-free survival or overall survival [59]. In a similar phase 2 trial involving trametinib, another MEK inhibitor, there was no survival benefit of the combination with docetaxel over docetaxel alone among previously treated patients with *KRAS*-mutant NSCLC [60].

On a biological level, the resistance to downstream blockage is likely due to a bypassing of the blocked pathway by the activation of alternative, parallel, RAS dependent pathways. Furthermore, it has been observed that MEK inhibition downregulates normal negative feedback mechanisms, inducing an upregulation of receptor tyrosine kinases (RTKs) upstream [61]. This mirrors a resistance mechanism observe when targeting *BRAF^V600E^* mutated cancers with direct BRAF inhibitors. Here, downregulating the negative feedback mechanisms causes EGFR-driven activation of parallel pathways including CRAF and RAS. This phenomenon has been studied intensely in the context of the aggressive *BRAF^V600E^* mutant subset of colorectal cancers. Upon targeting BRAF in these diseases, there is a higher level of EGFR than in melanoma cells [62]. To compensate this EGFR-mediated resistance, a recent approach has been to associate BRAF, MEK and EGFR inhibitors simultaneously in colorectal cancer, inducing a higher response and overall survival than standard therapy [63]. There is still significant room for improvement in this domain, but it shows that a better understanding of the mechanisms of resistance to therapy could allow physicians to ultimately tailor the treatment to extend the benefit for patients.

The loss of wild-type *KRAS* also plays a role in the sensitivity to MEK targeting agents in KRAS-mutant cell lines. Wild-type KRAS appears to promote resistance to MEK inhibition, possibly due to its tendency to form dimers with mutant KRAS [64]. Dimerisation is a necessary step in the activation of KRAS, including oncogenic signalling. Therefore, the loss of wild-type KRAS and subsequent decrease in dimerisation between it and the mutated variants may play a role in preventing the carcinogenic potential and overall function of mutant KRAS [64].

While KRAS G12C inhibition is recent, the emergence of acquired resistance has already been documented. In preclinical models, *KRAS* G12C mutant lung adenocarcinoma cell lines treated with the ARS-1620 inhibitor displayed varying degrees of MAPK pathway reactivation. As we discussed previously with regards to inhibitors of downstream Ras signalling, targeting the KRAS RTK itself was effective in some cell lines, while targeting the PI3K pathway directly provided greater inhibition in others [65]. The heterogeneous efficacy of KRAS G12C inhibition by ARS-1620 is explained by the identification of distinct subgroups within the cell lines, each with their own response upon exposure to the drug. Most cell lines became quiescent, entering the G0 state when treated with ARS-1620. However, some rapidly regained their RAS signalling activity and restarted proliferating. This RAS signalling reactivation appears to be the result of novel KRAS G12C production, stemming from the reduction in MAPK signalling. The newly formed KRAS G12C remains in its activated, GTP bound form thanks to the influence of EGFR and SHP2 signalling. In this state, it is insensitive to KRAS G12C blocking drugs. In order for KRAS G12C to escape from the quiescent, drug-induced G0 state, Aurora kinase A (AURKA) and the downstream CRAF also play a role by stabilising active KRAS [54].

While the production of new active KRAS G12C appears to be implicated in resistance to targeted therapy and persistent cell proliferation, another possibility is an adaptive wild-type RAS response to G12C inhibition. Under pressure from KRAS G12C inhibitors, a feedback loop can stimulate RTKs, leading to the activation of HRAS and NRAS, and ultimately, independent KRAS G12C signalling. On a biological level, it appears as though no single RTK activity was required for signalling in every KRAS G12C model. However, the co-inhibition of the SHP2 phosphatase had a broad efficacy in all models and led to the inhibition of the above-mentioned feedback reactivation mechanism [66]. SHP2 plays a role in mediating proliferative signalling between a number RTKs and the RAS pathway. Therefore, it has become an attractive target for combination therapies with KRAS G12C inhibitors to attempt to increase both primary efficacy and duration of response. This combination is underway in a phase I/II clinical trial with adagrasib (ClinicalTrials.gov Identifier NCT04330664), and other early phase trials are also exploring SHP2 inhibitors. Both RTK and SHP2 appear to play a significant role in acquired resistance to KRAS G12C inhibition and the best approach to targeting these is yet to be clear [67].

## 5. Resistance to KRAS^G12C^ Inhibitors in Patients

The precise mechanism of resistance in patients with cancer treated with KRAS G12C inhibitor is unclear at the moment. It is likely that intrinsic and acquired resistance may co-exist and be intertwined in the same patient treated with KRAS^G12C^-targeted therapies [45]. A number of co-occurring mutations may contribute to adaptive resistance across *KRAS* mutant cancers [51]. Molecular alterations, such as *TP53* (tumor protein p53), *CDKN2A* (cyclin-dependent kinase inhibitor 2A), *STK11* (serine/threonine kinase 11), KEAP1 (Kelch-like ECH-associated protein 1), might play an important role to explain the heterogeneity of response, however limited data are available on their role in predicting adaptive resistance [55]. The presence of co-occurring mutations, namely *KEAP1* or *STK11*, could affect the efficacy of the KRAS G12C inhibitor, sotorasib. Strong conclusions cannot be drawn due to the small number of patients analysed in the Codebreak100 tria [43].

Recently, Tanaka et al. [68] described a patients with KRAS G12C-mutant NSCLC who developed polyclonal acquired resistance to adagrasib with the emergence of 10 heterogeneous resistance alterations in serial cell-free DNA. The alterations spanned four genes (*KRAS, NRAS, BRAF, MAP2K1*), all of which converge to reactivate RAS-MAPK signalling. They identified a de-novo KRAS Y96D mutation affecting the switch-II pocket, able to interfere with key protein-drug interactions and confer resistance to KRAS-G12C KIs in engineered and patient-derived KRAS G12C cancer models. Interestingly, a novel, functionally distinct tri-complex KRAS G12C active-state inhibitor, RM-018 retained the ability to bind and inhibit KRAS G12C/Y96D and could overcome resistance.

An Important contribution to the understanding of resistance mechanisms was provided by Koga et al., [69] who generated 142 Ba/F3 clones resistant to either sotorasib or adagrasib. Thereof, 124 (87%) harboured secondary *KRAS* mutations, comprising 12 distinct *KRAS* mutations including Y96D/S, resistant to both inhibitors. The combination of a novel SOS1 inhibitor, BI-3406, and trametinib showed promising activity against this specific acquired resistance. Furthermore, the G13D, R68M and A59S/T mutations were highly resistant to sotorasib but remained sensitive to adagrasib. Conversely, KRAS Q99L was resistant to adagrasib but sensitive to sotorasib.

Genomic analyses including co-existing alterations should be documented in future therapies and trials to optimize treatment choices. Several studies are ongoing combining KRAS G12C inhibitors with other compounds (Table 1) to overcome resistance and improve clinical benefit.

## 6. How to Overcome Vertical Signal Resistance Pathways

Targeting KRAS induces a reduction in the ERK-mediated negative feedback loop, thus upregulating RTK expression. This upregulation then participates in reactivating new RAS signalling, promoting cell proliferation through SHP2 mediated RAS activation, in spite of upstream drug-induced KRAS inhibition [70]. The RAS reactivation’s dependency on SHP2 suggests that combining a KRAS and SHP2 inhibitor could lead to greater anti-proliferative efficacy [54]. In preclinical models, this has been tested. Using afatinib or erlotinib to prime cells potentiated the subsequent efficacy of the KRAS G12C inhibitor, ARS-853 [39,40]. Concurrent fibroblast growth factor receptor (FGFR) inhibitors or inhibitions of tyrosine-protein kinase Met (c-MET), were found to be more effective in inhibiting cell growth in vitro, compared to targeting KRAS G12C alone [71].

The potential synergy of targeting multiple RTKs to improve RAS inhibition is inconsistent in different preclinical models. It also highlights the challenge of knowing which RTK plays a significant role in RAS reactivation under therapeutic pressure [55]. Furthermore, RTK-associated phosphatase SHP2 might represent a possible targetable RTK signalling node and has been explored [55]. RTKs activates RAS in non-tumoral cells by recruiting the SHC-GRB2-SOS1 complex independently of SHP2 [72]. In cancer cells harbouring *KRAS G12C* mutations, SHP2 inhibition triggers a senescence response in vivo and in growth factor-limited conditions [73]. In melanoma, targeting RAS downstream effectors with MEK inhibitors on their own triggers the relief of ERK-mediated feedback inhibition of RTK signalling. This causes RAS reactivation which is SHP2 dependent [74]. The mechanism through which SHP2 activates RAS is still unclear. Combining KRAS G12C and SHP2 inhibitors has demonstrated improved efficacy in preclinical and animal models [54]. Similarly, in another preclinical model, the use of the KRAS KI, adagrasib, combined with RMC-4550, a direct SHP2 inhibitor, improved the inhibition of RAS signalling and anti-proliferative efficacy compared to adagrasib alone. Perhaps what is most interesting about this approach is that the increased efficacy was detected in models sensitive to adagrasib, as well as rendering refractory models sensitive to the combination therapy [75]. Of course, as with all therapeutic combinations, toxicity could be a limiting factor for use in patients.

Another possible target is SOS1, the guanine nucleotide exchange factor that activates KRAS [76]. Some SOS1 inhibitors have been investigated and BAY-29 showed high efficacy once combined with ARS-853, a KRAS G12C inhibitor, to inhibit Ras activation and cell proliferation by disrupting RAS-SOS1 interactions [77].

A step further to overcome resistance and increase treatment efficacy may involve upstream targeting. While it would not induce specific inhibition of steps of the downstream pathway, it would limit the activation of parallel signalling pathway, likely resulting in fewer side effects and more tolerable treatment [78]. Blocking any of a number of signalling pathways including PI3K, EGFR and FGFR appears to provide a synergistic anti-proliferative effect with KRAS G12C inhibition in vitro [39,65]. The biological rationale behind the potential efficacy of a combination of a KRAS G12C inhibitor and PI3K inhibitor is that they could decrease phosphatidylinositol (3,4,5)-trisphosphate (PIP3)-bound GAB adaptor proteins. The latter are involved in ERK reactivation, thus by decreasing both the ERK and PI3K signalling cascades, cell growth inhibition could be potentiated [65]. Lee et al. published an experiment pertaining to a potential new therapeutic combination involving a KRAS G12C inhibitor, ARS-1620, and alisertib, an AURKA inhibitor. The latter has been shown to overcome resistance to KRAS inhibition is some ARS-1620 refractory tumours [54]. AURKA is involved in the regulation of cell cycles. It is a mitotic serine/threonine kinase that exerts its inhibitory effect by blocking the interactions between CRAF and KRAS, suppressing ERK signalling, and inhibiting cell growth [54].

## 7. Combining Current Treatments in KRAS G12C Mutant Cancers

Chemotherapy remains an integral treatment for patients with cancer, in particular lung cancer. It is routinely associated with immune checkpoint inhibitors in first-line [6] It is therefore of interest to explore whether the combined use of standard-of-care chemotherapy or immunotherapy with a KRAS G12C inhibitor could be synergistic. In advanced KRAS G12C mutant NSCLC, sotorasib and adagrasib have been assessed administered concomitantly with carboplatin or palbociclib, [75,79]. The combination of carboplatin and sotorasib resulted in significant tumour regression in xenograft mouse models [75,79]. Similarly, adagrasib combined with palbociclib, a CDK4/6 inhibitor approved in breast cancer, showed anti-proliferative effects in adagrasib-resistant models. The authors hypothesize that curbing the retinoblastoma protein (Rb)/E2F transcription factor (E2F) signalling [75] explains the efficacy of this combination.

In vitro data suggest that *KRAS* mutant cancers are immunosuppressive, as oncogenic KRAS signalling can induce the expression of immunomodulatory factors [80]. It is likely that KRAS inhibition could convert the immunosuppressive tumour microenvironment into one that favours antitumor immune responses. Treatment with high dose sotorasib showed durable tumour regression in immunocompetent mice. On the contrary, in immunocompromised mice, tumours rapidly progressed after a short response [79]. In KRAS G12C models, similar pre-clinical data were seen with adagrasib, which enhances antigen presentation, and stimulates the tumour immune microenvironment [81].

## 8. Conclusions

Cancers harbouring *KRAS* mutations comprise a very heterogeneous selection. Both the biology of *KRAS*-driven diseases and their sensitivity to small molecule KIs are influenced by the type of KRAS variant and presence of co-mutations. Recently, sotorasib and adagrasib have shown clinical activity in NSCLC. Their efficacy is unprecedented for KRAS G12C targeting agents; however, enthusiasm must be tempered by resistance mechanisms. Both upstream and downstream strategies have been explored to overcome this resistance and enhance the efficacy of KRAS G12C inhibitors. Off target therapy like chemotherapy might prove to be a fruitful combination with these KIs. Similarly combining KRAS inhibitors with immune checkpoint inhibitors could improve their efficacy by modulating the tumour microenvironment and increased the sensitivity to checkpoint inhibitors. Potential co-mutations known to affect the immune microenvironment, such as *STK11/KEAP1*, are known to reduce the benefit derived from immune checkpoint inhibitors in KRAS-mutant NSCLC. Their role, and that of other concurrent mutations including TP53, remains unclear and further studies are needed to clarify their prognostic and or predictive role in KRAS inhibition.

Finally, while the therapeutic landscape has already dramatically changed, combination trials are ongoing. Understanding the biology behind resistance mechanisms is the best way forward to optimize therapies offered to patients.

## Figures and Tables

**Table 1:** TKIs KRAS G12C Inhibitors in Clinical Development.

Drug	Sponsor	Stage	ClinicalTrials.gov Identifier
Sotorasib	Amgen	Phase I–III	NCT04185883
Adagrasib	Mirati	Phase I–III	NCT03785249
GDC-6036	Genentech/Roche	Phase I	NCT04449874
JNJ-74699157	Janssen	Phase I	NCT04006301
D-1553	Inventis Bio	Phase I	NCT04585035
